# Optogenetic Monitoring of Synaptic Activity with Genetically Encoded Voltage Indicators

**DOI:** 10.3389/fnsyn.2016.00022

**Published:** 2016-08-05

**Authors:** Ryuichi Nakajima, Arong Jung, Bong-June Yoon, Bradley J. Baker

**Affiliations:** ^1^Center for Functional Connectomics, Korea Institute of Science and TechnologySeongbuk-gu, Seoul, South Korea; ^2^College of Life Sciences and Biotechnology, Korea UniversitySeongbuk-gu, Seoul, South Korea; ^3^Department of Neuroscience, Korea University of Science and TechnologyDaejeon, South Korea

**Keywords:** genetically-encoded voltage indicators, synaptic activity, optogenetics, brain slices, *in vivo*

## Abstract

The age of genetically encoded voltage indicators (GEVIs) has matured to the point that changes in membrane potential can now be observed optically *in vivo*. Improving the signal size and speed of these voltage sensors has been the primary driving forces during this maturation process. As a result, there is a wide range of probes using different voltage detecting mechanisms and fluorescent reporters. As the use of these probes transitions from optically reporting membrane potential in single, cultured cells to imaging populations of cells in slice and/or *in vivo*, a new challenge emerges—optically resolving the different types of neuronal activity. While improvements in speed and signal size are still needed, optimizing the voltage range and the subcellular expression (i.e., soma only) of the probe are becoming more important. In this review, we will examine the ability of recently developed probes to report synaptic activity in slice and *in vivo*. The voltage-sensing fluorescent protein (VSFP) family of voltage sensors, ArcLight, ASAP-1, and the rhodopsin family of probes are all good at reporting changes in membrane potential, but all have difficulty distinguishing subthreshold depolarizations from action potentials and detecting neuronal inhibition when imaging populations of cells. Finally, we will offer a few possible ways to improve the optical resolution of the various types of neuronal activities.

## Introduction

As developers of genetically-encoded voltage indicators (GEVIs) we are often asked for our best probe. Until recently, a good GEVI would have been any that gave a voltage-dependent, optical signal in mammalian cells (Dimitrov et al., 2007; Lundby et al., 2008, 2010; Perron et al., 2009a,b). Now the experimenter has several probes to choose from that differ in their voltage-dependencies, speed, signal size, and brightness (Akemann et al., 2012; Jin et al., 2012; Kralj et al., 2012; Han et al., 2013; St-Pierre et al., 2014; Zou et al., 2014; Gong et al., 2015; Piao et al., 2015; Abdelfattah et al., 2016). The combinations of these varying characteristics result in strengths and weaknesses of every GEVI available. There is no perfect probe that can optically resolve action potentials, synaptic activity, and neuronal inhibition *in vivo*. Some GEVIs will give large, voltage-dependent optical signals but are very dim limiting their usefulness *in vivo*. Others will give large optical signals but are very slow reducing their ability to resolve fast firing action potentials. So now, when asked which is the best probe, the answer is simply another question. What do you want to measure? To fit with the theme of this edition, we will assume that the answer to that question is synaptic activity.

Several reviews have been published comparing the signal size, speed, and brightness of the GEVIs currently available at the time of publication (Wachowiak and Knöpfel, 2009; Akemann et al., 2012, 2015; Knöpfel, 2012; Mutoh et al., 2012; Perron et al., 2012; Mutoh and Knöpfel, 2013; Emiliani et al., 2015; Knöpfel et al., 2015; St-Pierre et al., 2015; Storace et al., 2015b, 2016; Antic et al., 2016). In this review, we will shift the focus to one of the lesser considered characteristics of a GEVI, the voltage-sensitivity of the probe. Of course, the other characteristics, especially signal size and brightness, are still important, but the range and steepness of the voltage sensitivity of the optical response have extremely important consequences on which type of neuronal activity a GEVI reports well. For instance, a GEVI with a voltage range from −20 mV to +30 mV would be perfect for monitoring action potentials but not ideal for observing synaptic potentials. Even the shape of the slope of the optical response over the voltage range of the probe will affect its performance. The consequences of the slope and voltage range should also be considered when choosing probes for monitoring neuronal activity.

## A Brief Description of Currently Available GEVIs

There now exist several GEVIs with multiple mechanisms of converting membrane potential changes into an optical signal. These GEVIs fall into two main classes. One class utilizes bacterial rhodopsin to detect alterations in voltage, while the other class relies on a voltage-sensing domain (VSD) from voltage-sensing proteins. Another viable alternative for optical, neuronal recordings is hybrid voltage sensor (hVOS) which consists of a genetically encoded component, a farnesylated fluorescent protein (FP), and a quenching compound, dipicrylamine (DPA; Chanda et al., 2005; Wang et al., 2010, 2012; Ghitani et al., 2015). The requirement for the treatment with an exogenous chemical limits hVOS use *in vivo* but still has value for imaging voltage in slice preparations.

**Figure 1:**
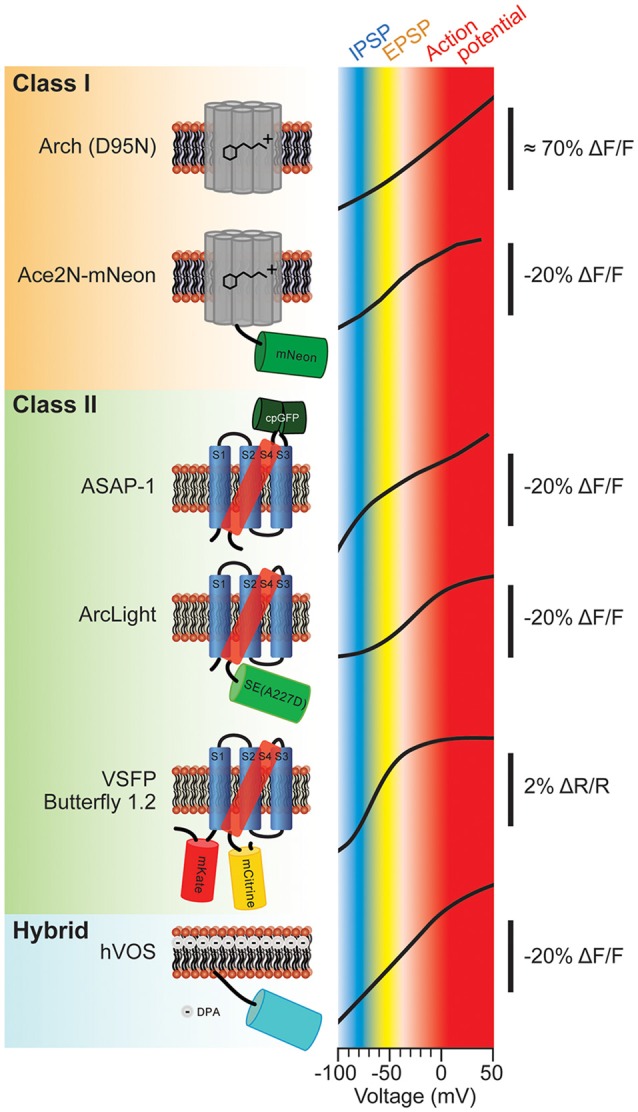
**Various types of genetically-encoded voltage indicators (GEVIs) and their voltage sensitivities.** The voltage sensitivities of different GEVIs are compared. Typical voltage ranges of inhibitory postsynaptic potential (IPSP), excitatory postsynaptic potential (EPSP) and action potential are indicated as blue, yellow and red, respectively. The vertical scale bar with minus ΔF/F indicates that the fluorescence dims upon depolarization of the plasma membrane. The voltage-sensitivity curves were as reported in: Arch (D95N; modified with permision from Kralj et al., 2012, Figures 3B, 5); Ace2N-mNeon (modified with permission from Gong et al., 2015, Figure 1D); ASAP-1 (modified with permission from St-Pierre et al., 2014, Figure 1D); ArcLight (modified with permission from Jin et al., 2012, Figure 1C); Butterfly 1.2 (modified with permission from Akemann et al., 2012, Figure 2C); hybrid voltage sensor (hVOS; modified with permission from Chanda et al., 2005, Figure 1D).

The molecular schematics of representative probes from these classes and their corresponding voltage ranges are shown in Figure 1. The voltage range of a generic mammalian neuron is color coded to represent different neuronal activities. The inhibitory postsynaptic potential (IPSP) voltage range is shown in blue. The excitatory postsynaptic potential (EPSP) voltage range is shown in yellow. Voltages corresponding to action potentials are color coded red. As can be seen from Figure 1, the slopes of these optical voltage responses are significantly different. This is an important consideration when measuring synaptic potentials. For instance, Butterfly 1.2 has nearly reached its maximal fluorescent change at −40 mV which would imply that differentiating subthreshold potentials from action potentials will be very difficult.

### Class I—The Rhodopsin-Based Probes

Channel rhodopsin has revolutionized neuroscience. The rhodopsin-based voltage sensors are promising to do the same thing for imaging membrane potential. First developed in Adam Cohen’s lab, the intrinsic fluorescence of rhodopsin as a Schiff base being protonated or deprotonated in response to voltage was used to image changes in membrane potential (Maclaurin et al., 2013). This probe, Arch, was extremely fast having a tau under 1 ms. The fast optical response is due in part to the fact that the chromophore resides in the voltage field enabling a nearly instantaneous response. The signal size was also large giving roughly a 70% ΔF/F optical signal per 100 mV membrane depolarization (Kralj et al., 2012; Figure 1, Arch (D95N)).

Arch excelled in speed and signal size but suffered from some serious weaknesses. The first weakness was that the original version had an associated, light-induced current. The D95N mutation drastically reduced this current but also resulted in a slower probe (Kralj et al., 2012). The second weakness was that it does not traffic well to the plasma membrane. Even with the addition of endoplasmic reticulum and Golgi network release motifs, every image of a rhodopsin probe in the literature exhibits high intracellular fluorescence (Kralj et al., 2012; Flytzanis et al., 2014; Gong et al., 2014, 2015; Hochbaum et al., 2014; Hou et al., 2014). The third and most devastating weakness was that Arch is very dim. The best versions of Arch and related probes are still at least 5× dimmer than the green fluorescent protein (GFP) requiring exceptionally strong illumination, at least 700× the light intensity required for ASAP-1 to visualize the probe activity (Flytzanis et al., 2014; St-Pierre et al., 2014).

**Figure 2:**
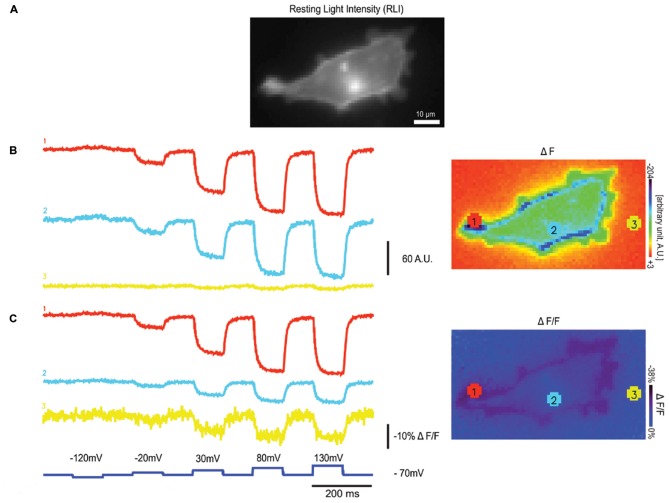
**Varying light levels affect ΔF and ΔF/F values. (A)** An HEK 293 cell expressing a single fluorescent protein (FP) based GEVI, Bongwoori, is shown in resting light intensity (RLI), **(B)** the fluorescence traces of averaged ΔF (F_x_-F_0_) values from three different regions with varying intensity, **(C)** the fluorescence traces showing averaged ΔF/F values from the same regions in (**B**; modified with permission from Lee et al., 2016, Figure 6).

The weak fluorescence of Arch limits its use to single cell in culture studies or to *C. elegans* (Kralj et al., 2012; Flytzanis et al., 2014) for two main reasons. The first is that the intrinsic fluorescence of higher order neuro-systems will mask the fluorescence of Arch-type probes. The second is that ΔF in addition to ΔF/F is an important characteristic of the GEVI when it comes to the signal to noise ratio. An example of this is shown in Figure 2. The HEK cell in Figure 2 is expressing a GEVI from which the ΔF and the ΔF/F traces from three different light levels are shown (Lee et al., 2016). As can be seen from this comparison, a high ΔF/F value can be achieved by a large change in fluorescence or a small change in fluorescence when the probe is dim. Notice the increased noise in trace 3, a telltale sign of poor expression/dim fluorescence.

An ingenious solution to compensate for the poor fluorescence of the rhodopsin voltage probes was developed simultaneously by the Adam Cohen and Mark Schnitzer laboratories. By fusing an FP to the rhodopsin protein, förster resonance energy transfer (FRET) enabled the rhodopsin chromophore to affect the fluorescence of the fused FP. This design reduced the excitation light intensity needed to visualize the GEVI while maintaining the speed of the optical response since the voltage-sensing chromophore was still in the voltage field. These probes could also cover different wavelengths since many different FPs could be fused to rhodopsin and give a signal (Gong et al., 2014; Zou et al., 2014). While this made the rhodopsin probes better, the optical signal sometimes could only indirectly report neuronal activity by determining the frequency of the noise in the optical recording (See Supplementary Figure 5 in Gong et al., 2014). Now, an exciting new version using the FP, mNeonGreen, has recently been reported (Gong et al., 2015). mNeonGreen is a very bright FP (Shaner et al., 2013) enabling Ace2N-mNeon to resolve action potentials *in vivo* in both flies and mice.

### Class II—VSD Containing GEVIs

The second class of GEVIs is also the oldest. The original GEVI, Flash (Siegel and Isacoff, 1997), was the result of inserting GFP downstream of the pore domain of the voltage-gated potassium channel, Shaker. Like the rhodopsin-based probes, the first generation of VSD-based probes had significant drawbacks making them useless in mammalian cells (Baker et al., 2007). The main problem was that the GEVIs did not traffic to the plasma membrane. In 2007, one of the biggest advancements in GEVI development was achieved by the Knöpfel laboratory when they fused FPs to the VSD of the voltage-sensing phosphatase gene from *Ciona intestinalis* (Murata et al., 2005). This probe, voltage-sensing fluorescent protein (VSFP) 2.1 trafficked well to the plasma membrane which resulted in the first voltage-dependent optical signals from cultured neurons (Dimitrov et al., 2007).

Another issue with VSD-based GEVIs is that the chromophore resides outside of the voltage field so the optical signal relies on the conformational change of the VSD. These probes are therefore generally slower than the rhodopsin-based probes, but a recently developed red-shifted GEVI is extremely fast having taus under 1 ms (Abdelfattah et al., 2016).

There are three different designs for GEVIs that utilize a VSD. The first design uses a FRET pair flanking the VSD. An example is Butterfly 1.2 (Akemann et al., 2012). This probe is somewhat slow and gives a very small optical signal, less than 3% ΔF/F per 100 mV depolarization. A butterfly style probe that gives a faster and larger optical signal was developed last year called Nabi (Sung et al., 2015). An advantage of FRET-based probes is that the ratiometric imaging can remove movement artifacts due to respiration and blood flow *in vivo*. Theoretically, a ratiometric measurement could also be used to determine the absolute value of the membrane potential since the ratio is concentration independent. In practice, however, the relative fluorescence of the two chromophores differ substantially resulting in a potential increase in the noise for the analysis of the optical signal. Often the experimenter should only analyze the brighter signal (Wilt et al., 2013). It is also difficult to only excite the donor chromophore and not the acceptor as well. These factors combined with the relatively low signal size of FRET-based probes prohibit any reliable absolute measurement of membrane potential.

The second design involves a circularly-permuted fluorescent protein (cpFP) attached to the VSD. Initial designs fused the cpFP downstream of the VSD so that the chromophore was in the cytoplasm (Gautam et al., 2009; Barnett et al., 2012). Electrik PK gave very small signals less than 1% ΔF/F per 100 mV depolarization but were very fast having a tau under 2 ms. A substantial increase in signal size was achieved when the cpFP was placed between the S3 transmembrane segment and the S4 transmembrane segment of the VSD putting the chromophore outside of the cell (St-Pierre et al., 2014). This probe, ASAP-1, is one of the better GEVIs giving a fast and robust optical signal (tau = 1–2 ms and about 20% ΔF/F per 100 mV depolarization in HEK cells). ASAP-1 has a very broad voltage range which is virtually linear over much of the physiologically relevant potentials of neurons.

The third design of GEVIs that utilize a VSD simply fuses the FP at the carboxy-terminus which puts the chromophore in the cytoplasm. During a systematic test of different FPs fused at different linker lengths from the VSD done in collaboration by Vincent Pieribone’s lab and Larry Cohen’s lab, a point mutation on the outside of the FP, Super Ecliptic pHlorin (Miesenbock et al., 1998; Ng et al., 2002) converted an alanine to an aspartic acid improving the optical signal 15 fold from 1% ΔF/F to 15% per 100 mV depolarization of the plasma membrane (Jin et al., 2012). This negative charge on the outside of the β-can seems to affect the fluorescence of a neighboring chromophore when S4 moves since mutations that favor the monomeric form of the FP reduce the voltage-dependent optical signal substantially (Kang and Baker, 2016). Further development of ArcLight has gotten signals as high as 40% ΔF/F per 100 mV depolarization step (Han et al., 2013). While ArcLight has the drawback of being slow, its brightness and signal size make it one of the better probes for imaging *in vivo* and in slice. In 2015, two publications improving the speed of this sensor were published. One dramatically improved the off rate called Arclightening but reduced the signal size to under 10% ΔF/F per 100 mV depolarization (Treger et al., 2015). The other, Bongwoori, improved the speed of the sensor and shifted the voltage response to more positive potentials which improved the resolution of action potentials but decreased the signal size for synaptic potentials (Piao et al., 2015). The reduced optical signal response for sub-threshold potentials gives Bongwoori a better “contrast” for optically resolving action potentials.

A final design for researchers to consider when choosing a GEVI is the genetically encoded, hVOS (Chanda et al., 2005; Wang et al., 2010, 2012; Ghitani et al., 2015). First developed in the Bezanilla lab, hVOS consists of an FP anchored to the plasma membrane with the addition of a small charged molecule, DPA, that binds to the plasma membrane effectively acting as a fluorescent quencher. Since DPA is a lipophilic anion, the quenching agent will move from the outer surface of the plasma membrane to the inner surface upon membrane depolarizations generating a voltage-responsive fluorescent signal. Like the other sensors, hVOS also has drawbacks which are primarily due to the fact that an exogenous chemical must be administered to the sample to be imaged. This is not a trivial process since too much DPA will significantly increase the capacitance of the plasma membrane and alter the neuronal activity of the cell. However, once the appropriate conditions are determined, hVOS gives optical signals for subthreshold potentials as well as action potentials in slice from populations of cells (Wang et al., 2012) or individual cells when expression of the FP is sparser (Ghitani et al., 2015).

## Synaptic Activity Monitoring in Slices with GEVIs

Brain slices are invaluable for studying in detail the cellular, molecular, and circuitry activity of neuronal functions (Ting et al., 2014). GEVIs can expand this information since every pixel potentially becomes an electrode. There are not many examples of synaptic potential recordings from GEVIs in slice. Most examples are proof-of-principle type of recordings in the original publication of a new sensor to demonstrate its potential. The VSFP family of GEVIs are the most published recordings in brain slice (Akemann et al., 2013; Scott et al., 2014; Carandini et al., 2015; Empson et al., 2015; Mutoh et al., 2015). Here, we compare optical synaptic recordings in brain slices from VSFP Butterfly 1.2 and hVOS.

### FRET Signals of Butterfly in Cortical Brain Slices

**Figure 3:**
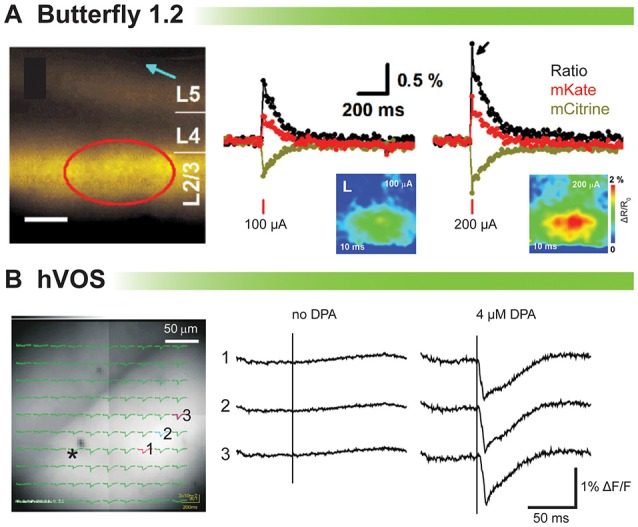
**Comparison of voltage indicators for synaptic imaging in brain slices. (A)** Fluorescence and ratiometric signals of voltage-sensing fluorescent protein (VSFP) Butterfly 1.2 in cortical brain slices (modified with persmission from Akemann et al., 2012, Figures 3I,J). Left: wide-field fluorescence image with indicated position of the stimulation electrode (blue arrow). Scale bar, 150 μm. Middle and right: a single-pulse synaptic stimulus (middle: 100 μA; right: 200 μA) induced a depolarizing response as indicated by a transient decrease in mCitrine (yellow) and increase in mKate2 (red) emission from the pixels indicated by a red circle. The ratio of the two emission spectra is in black. **(B)** Stimulus-evoked fluorescence changes (ΔF/F) and their dipicrylamine (DPA) dependence (modified with permission from Wang et al., 2012, Figure 3). A slice expressing hVOS 1.5 from mouse hippocampus. All recordings were from the striatum-radiatum (sr) of the CA1 region. Left: a slice from hVOS 1.5 line 602 with images and fluorescence traces superimposed. Traces are from the three numbered locations before and after addition of DPA. The stimulation site is indicated by the asterisk. All traces of hVOS signals are averages of 10 trials.

Figure 3A shows the population imaging in coronal cortical slices prepared from a mouse brain electroporated *in utero* with VSFP-Butterfly 1.2 (Akemann et al., 2012). To explore voltage imaging from populations of cells, cortical slices were imaged at low magnification while delivering a single electrical stimulus (Figure 3A, left panel). The amplitude of the evoked optical signal ranged from 1 to 1.5% ΔR/R_0_ (Figure 3A). Disinhibition with 25 mM gabazine increased the signal to 11% ΔR/R_0_ (Akemann et al., 2012). Since VSFP-Butterfly 1.2 is a FRET probe, the ratio of the fluorescent change can be reported, but in slice the advantage of a ratiometric recording is of lesser value since movement artifacts due to respiration and blood flow do not exist. Despite this advantage, the voltage-dependent change in fluorescence is quite small, less than 0.5% ΔF/F which requires multiple trials to improve the signal to noise ratio.

### hVOS Signal in the Hippocampal Slice

Figure 3B shows the hVOS signal in a hippocampal slice. The electrical stimulation evoked clear fluorescence changes only when 4 μM DPA was present. This concentration of DPA provided excellent signal up to 2 h with minimum pharmacological action (Wang et al., 2010). The hVOS probe fluorescence decreases with membrane depolarization because DPA moves to the inner surface of the cell membrane where hVOS probes are anchored; the arrival of DPA quenches the probe fluorescence. Responses of approximately 1–3% could be seen throughout the field of view (Wang et al., 2012).

VSFP-Butterfly 1.2, and hVOS can all generate an optical signal corresponding to synaptic responses in acute brain slices. hVOS has the larger ΔF/F. VSFP-Butterfly 1.2 does not require additional drug application to detect voltage changes in the neuron. Other sensors can also give optical signals in slice but those recordings have focused on action potentials in individual cells and are not shown here. The brightness, signal size, and voltage range of ASAP-1 make it a potentially useful sensor for imaging synaptic potentials in slice. While there are no reports in the literature of ArcLight being used to analyze neuronal activity in brain slices, the brightness, signal size and voltage-sensitivity are also ideal for optically recording synaptic potentials.

## Synaptic Activity Monitoring *In Vivo* with GEVIs

While slice recordings are extremely valuable for deciphering neuronal circuitry, the ultimate goal of voltage imaging is to detect neuronal activity in a behaving animal. This is an ambitious endeavor with very few examples, but some GEVIs are now capable of giving a robust signal that allows *in vivo* imaging.

**Figure 4:**
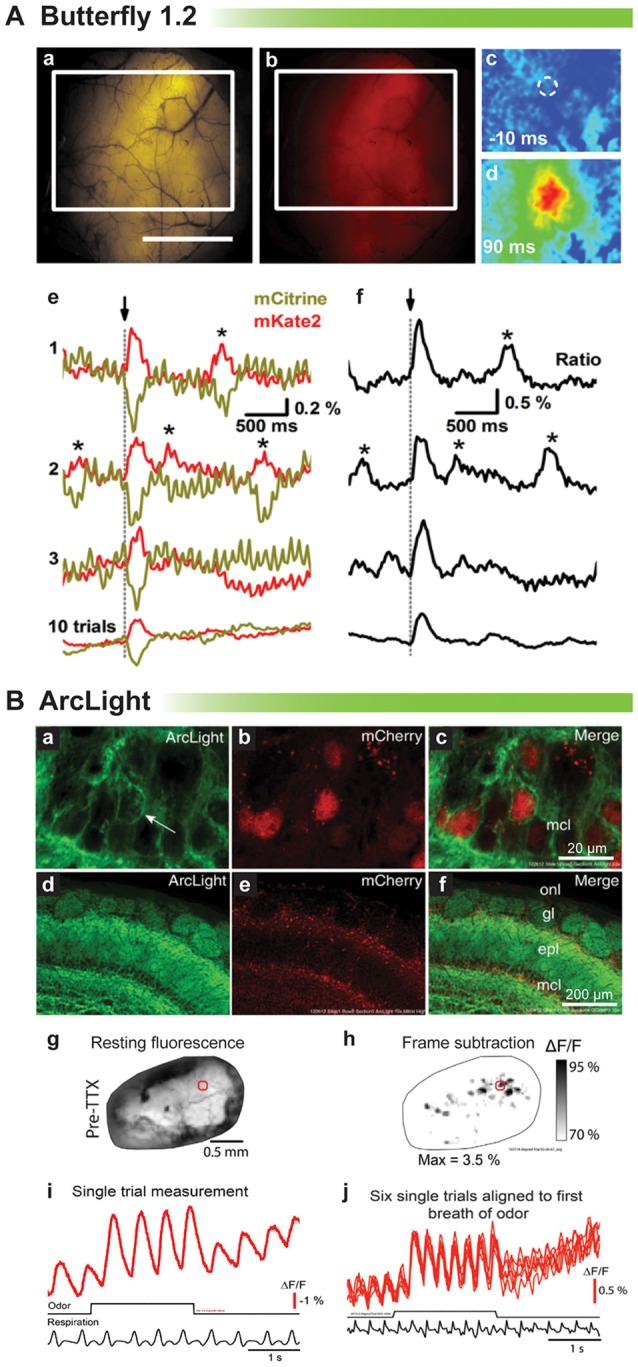
**Comparison of voltage indicators for synaptic imaging *in vivo*. (A)** Butterfly 1.2 signal in the somatosensory cortex during whisker stimulation (modified with permission from Akemann et al., 2012, Figures 6B,D,E). **(a,b)** Fluorescence image of mCitrine **(a)** and mKate2 **(b)** from a mouse expressing VSFP-Butterfly 1.2. White rectangle indicates area imaged in **(c)**. Scale bar, 2 mm. **(c,d)** Ratio images obtained at times before and after brief deflection of the D1 whisker performed at time 0. **(c)** 10 ms before **(d)** 90 ms after the deflection. **(e)** Single-sweep (1, 2, and 3) mCitrine (yellow) and mKate2 (red) optical signals sampled from the region of interest indicated by white circle in **(c)**, together with the 10-trial average (bottom). Asterisks mark signals corresponding to potential spontaneously occurring voltage transients. **(f)** Ratio (ΔR/R_0_) signals corresponding to the traces in **(e)**. **(B)** Odor-evoked signals of ArcLight in olfactory bulb (modified with permission from Storace et al., 2015a, Figures 1, 2). **(a–c)** High magnification confocal images of ArcLight demonstrate membrane localization (arrow). The FP, mCherry, is localized to the nucleus to facilitate identification of transduced neurons. **(d–f)** Low magnification of the olfactory bulb—onl, olfactory nerve layer; gl, glomerular layer; epl, external plexiform layer; mcl, mitral cell layer. **(g)** Wide-field resting fluorescence intensity. **(h)** Glomerular patterns of activation after odor stimulation. **(i)** Odor-evoked optical signals from the region of interest marked with a red circle in **(g,h)**. **(j)** Six unfiltered single trials aligned to the first sniff of odorant.

Proof of principle for *in vivo* voltage imaging was established by the Knöpfel lab using the VSFP family of probes (Akemann et al., 2012, 2013). Figure 4A shows single trial responses in the barrel cortex during whisker stimulation. Clearly, a stimulus evoked voltage signal could be detected in single trials even though the signal size is very small. Asterisks denote potential spontaneous voltage transients. However, unlike the stimulus evoked optical response, these potential transients exhibit different start times and kinetics. Having a low signal to noise ratio undermines the confidence in reliably detecting neuronal activity trial to trial (Carandini et al., 2015). Another drawback with VSFP Butterfly 1.2 is that the V_1/2_ is roughly −70 mV with maximal fluorescent change occurring at −40 mV (Figure 1) making it virtually impossible to distinguish synaptic activity from action potentials based solely on signal size.

ArcLight has also been tested *in vivo* in mice and flies (Cao et al., 2013; Storace et al., 2015a). The V_1/2_ for ArcLight is around −30 mV making it ideal to detect neuronal activity in flies whose action potentials range from a resting potential of −40 mV to a final excitation of −10 mV. As a side note this is why our probe, Bongwoori, should not be used for imaging neuronal activity in flies since the V_1/2_ has been shifted to around 0 mV (Piao et al., 2015). Figure 4B shows a recording from a mouse expressing ArcLight in the olfactory bulb. As can be seen, respiration causes an optical artifact, but since ArcLight gives a large signal, identifying regions of the olfactory bulb responding to an odor is still possible. Again, though, it is not possible to resolve synaptic activity from action potentials.

**Figure 5:**
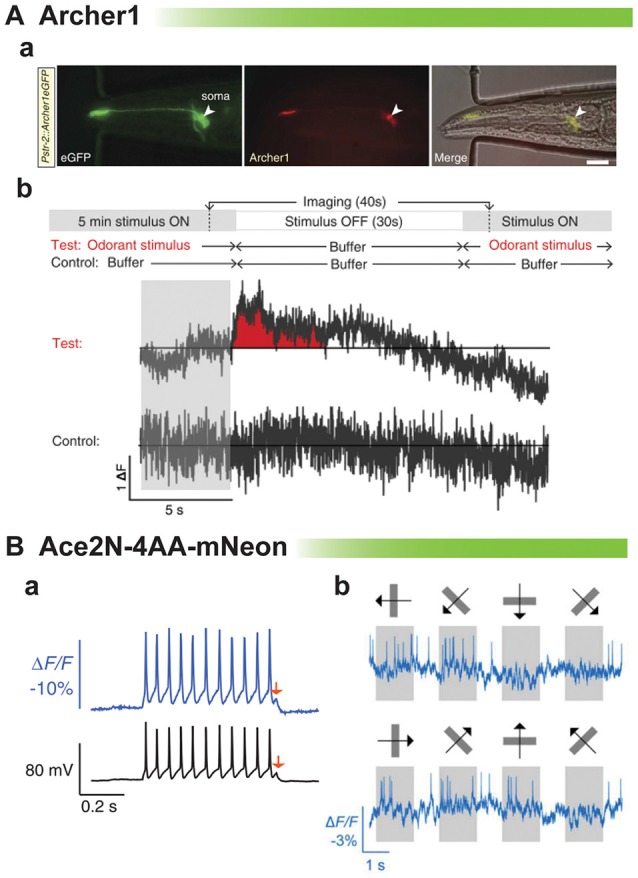
**Comparison of rhodopsin-based voltage indicators for synaptic imaging *in vivo*. (A)** Archer1 expressed in worms. **(a)** *C. elegans* expressing Archer1 shows fluorescence (λ = 655 nm; *I* = 880 mWmm^-2^, 100 ms exposure). Scale bar, 20 mm. **(b)** Top: experimental conditions: worms are stimulated with odorant (Isoamyl alcohol, IAA) for 5 min, flow is switched to buffer (S Basal) for 30 s, and then odorant flow is restored. The control conditions are performed on the same worm. Bottom traces: imaging of Archer1 fluorescence (250 Hz) (modified with permission from Flytzanis et al., 2014, Figures 5B,C). **(B)** Imaging single action potentials and subthreshold membrane voltage by Ace2N-4AA-mNeon (modified with permission from Gong et al., 2015, Figures 2A, 3D). **(a)** Optical resolution of action potentials of cultured hippocampal neurons under current clamp exhibiting best fit of optical data to electrical recording to date. Arrow denotes 5 mV depolarization. **(b)** Optical traces from a cortical V1→LM neuron in an awake mouse, showing visually evoked responses to drifting gratings.

The rhodopsin-based GEVIs have also been shown to elicit an optical signal *in vivo*. An example is shown in Figure 5A from the Gradinaru lab (Flytzanis et al., 2014). With the dim fluorescence of the GEVI, Archer, *C. elegans* is one of the few multicellular organisms one would be able to record from. After odorant stimulation, there is a slight variation in the ΔF compared to control. While there appears to be a slight signal, the low signal to noise ratio again undermines one’s confidence in being able to reliably detect neuronal activity from trial to trial.

The last example of *in vivo* recordings is the best example of resolving action potentials. Ace2N-4AA-mNeon has been imaged in flies and mice (Gong et al., 2015). This probe is extremely fast showing the best fit of optical data to voltage yet (Figure 5B). The red arrow shows an optical response to a 5 mV depolarization. However, comparing the optical signal at the spike to the subthreshold potential, one can see that the optical response is skewed towards action potential activity. This gives a fantastic response when imaging the visual cortex in response to visual stimuli. Action potentials are easily discernible. The ability to optically report synaptic activity is less clear but still promising.

## Conclusion

GEVIs come in many flavors. As demonstrated, the signal size, speed, and voltage sensitivity affect the neuronal activity a GEVI can resolve. Many probes will give an optical signal in slice and *in vivo* but some signals will be more informative. If the experimenter wants to image any neuronal activity from a population of cells in brain slice, the recommendations would be hVOS, ArcLight, and ASAP-1. All have a broad voltage range, traffic to the membrane well and give relatively large signals. ArcLight and ASAP-1 will have some difficulty in separating synaptic activity from action potentials due to their voltage sensitivities, but this could theoretically be overcome by co-expression of a red calcium sensor to verify action potential activity if the neuron tested has an action potential-induced calcium transient. If one wants to measure neuronal activity of individual cells in slice, then one should also consider Ace2N-4AA-mNeon.

Imaging single cells vs. a population of cells will also affect the choice of GEVI to be used. When imaging single cells, probes with broad voltage ranges will enable the optical detection of inhibition, synaptic potentials, and action potentials. However, these same probes when imaging large populations of cells are potentially less informative since the depolarization of a subgroup of neurons could swamp the small, hyperpolarizing signals from inhibited neurons.

**Figure 6:**
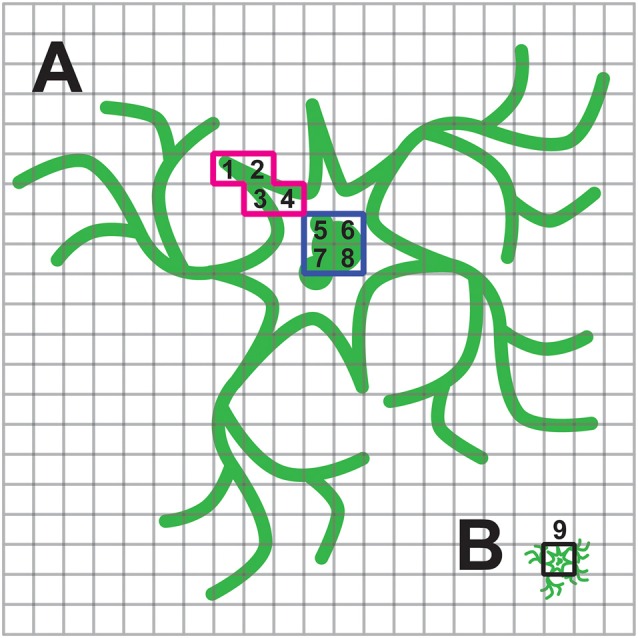
**Consequence of internal fluorescence when imaging population of cells. (A)** Schematic of a neuron under high magnification with plasma membrane and internal fluorescence in green. The cell is projected onto multiple pixels enabling the experimenter to choose pixels with optical activity. In this example the red, highlighted pixels labeled 1–4 have a large ΔF/F while pixels highlighted in blue, labeled 5–8, exhibit low or no change in fluorescence upon depolarization of the plasma membrane. **(B)** Same cell under low magnification now only projects onto a few pixels. The pixel highlighted in black, labeled 9, is a summation of pixels 1–8 in **(A)**. The experimenter is no longer able to avoid the non-responsive, internal fluorescence.

Inefficient trafficking or high intracellular expression will affect the voltage imaging of a population of cells more so than when imaging individual cells. The reason for this is that the spatial representation of the cell under high magnification onto the pixels of the camera has changed. Under high magnification, a researcher can choose only pixels that correspond to regions of the cell that exhibited a fluorescent response. When imaging a population of cells, a pixel will be less likely to capture only the responsive fluorescence. This situation is depicted in Figure 6. When imaging a single cell, it is much easier to avoid the internal, non-responsive fluorescence and maximize the signal to noise ratio.

While the GEVIs currently available have shown significant improvement in their ability to optically detect neuronal activity, there is still much room for improvement. Refining the voltage-sensitivity will enable maximizing the optical signal. For instance, a probe that only responded to hyperpolarization of the plasma membrane would make identifying the inhibited parts of a neuronal circuit much easier. Improving the membrane expression of the GEVI will decrease the nonresponsive fluorescence in a population of cells, thereby improving the signal to noise ratio. Most efforts to improve trafficking involve the addition of endoplasmic reticulum and Golgi release motifs. Codon optimization is another approach which for membrane proteins may be a misnomer. The idea of codon optimization is to use only the most abundant codons for rapid translation of the protein. This has been shown to be effective for cytoplasmic proteins. However, for membrane proteins slowing the translation to allow proper folding and insertion into the translocon may also be important (Norholm et al., 2012; Yu et al., 2015). Finally, limiting the expression of the GEVI to subcellular components (i.e., the soma, dendrites, etc.) could also focus the optical signal to the desired region of the neuron again improving the signal to noise ratio.

## Author Contributions

RN and AJ wrote the manuscript and contributed figures. B-JY and BJB helped to write the manuscript.

## Conflict of Interest Statement

The authors declare that the research was conducted in the absence of any commercial or financial relationships that could be construed as a potential conflict of interest.
